# Treatment of Enlargement of the Prostate

**Published:** 1901-11-30

**Authors:** 


### Hospital Clinics and Medical Progress.

### TREATMENT OF ENLARGEMENT OF THE PROSTATE.

In the second of the Harveian Lectures recently delivered, Mr. Buckston Browne dealt in great fulness with the important subject of enlargement of the prostate, laying stress more particularly upon the treatment to be adopted in cases of retention of urine arising from this cause. The first thing that he insists upon is the utmost care in the purification of catheters, in the cleansing of the glans penis, and in rendering the hands aseptic before passing any instrument for the relief of retention. Then, when catheterism becomes necessary in the case of an elderly man, it is always well to attend to the patient in his own warm room, while, if the patient be at all feeble, he should be kept altogether in bed for a few days. He also asserts most emphatically that there are no cases of prostatic disease where it is impossible to pass a catheter into the bladder. If a catheter will not pass readily in a case of prostatic retention, it will be because the forward curve of the urethra is too acute for the instrument to follow it, or because the point of the catheter has caught in one or other of the two prostatic sinuses on either side of the caput gallinaginis. When, therefore, the introduction of the catheter is arrested in these cases, the point impinges upon the posterior wall of the urethra or is caught in one of the pockets situated in that wall. It follows that for successful introduction the point of the catheter must hug the anterior wall of the urethra, and so the whole art of catheterising the prostatic urethra with soft instruments consists in making their points avoid the posterior wall. In successful prostatic catheterisation one of two things always occurs—either the catheter takes the form of the urethra or the urethra that of the catheter. In the first case the catheter must of necessity be a soft one. It is always well to begin with an indiarubber catheter. If this will not pass then the coudée catheter may be tried, keeping the beak well upwards all the way in. Then in point of usefulness comes the bicoudée catheter, which is a very efficacious instrument. The olivary catheter and the English gum catheter are rarely of use in cases of real difficulty. The value of the rubber and the coudée catheter may in certain cases be much enhanced by the use of a metal stylet of iron, lead, or silver. This will in many cases not require to be carried to the end of the catheter. If no soft catheter either with or without a stylet can be passed then we must make the urethra conform to the catheter; in other words we must use a silver instrument. "The very worst cases can always be relieved by a silver instrument if the patient be anæsthetised and if the surgeon guide the point of the instrument with his left forefinger in the rectum." A large No. 14 silver catheter with a short curve and fitted with a gum stylet is very useful. It is too large and blunt to catch in the prostatic sinuses and the short curve comes readily forward on depressing the shaft of the instrument, while the gum stylet is useful in preventing plugging of the catheter by blood clot. After the bladder has been emptied a soft catheter can always be passed in, if moulded on an iron stylet to the exact shape of the successful instrument, and tied in. The most grave and anxious cases are those where the retention has been allowed to become chronic, and in which the bladder is enlarged and distended. No surgeon should consent to treat such cases as out-patients. They must be sent to bed, and told that they will have to stay there for two or three weeks. In such cases catheterism must be commenced with every antiseptic detail, and the bladder should only be very gradually emptied, the catheter being passed every six hours and only, say, 17 ounces being drawn off each time, so that four or five days may pass before the bladder is completely emptied. If these largely distended bladders be suddenly emptied "there is almost sure within a few hours to be some hæmorrhage from the vesical veins which have been too suddenly relieved from a condition of chronic and severe pressure, the kidneys suffer severely from the shock, urinary fever follows, and the patient almost invariably dies," while, if they are only slowly emptied all may go well. If a prostatic patient be carefully introduced to the use of the catheter, and if he continue to use it with care and cleanliness, his prospects of life are good. A man dependent upon his catheter is by no means debarred from great activity, and instances are numerous of men, active in politics, law, medicine, and the church, distinguished in the work of scientific research, and even as sailors and sportsmen, who are in this condition. Still there are exceptional cases where simple catheterism will not alone suffice, or is impossible. Among such are those in which, hidden away but none the less irritating and torturing, there is a stone in the bladder. A prostatic case where the calls to empty the bladder are constant, and where, perhaps, the catheterism is difficult and painful, while the relief obtained is evanescent, and where no calculus can be found by ordinary methods of examination, should be kept at rest in a warm room, his catheter should be introduced rather too often than too seldom, the bladder should be washed out by mild solutions of nitrate of silver, boroglycerine, or glycerine and borax, and antiseptics and aperients should be administered by the mouth. Under such treatment, if the case be a simple one, the improvement which takes place is often astounding, and may be permanent if proper care be continued. But if improvement do not take place under such treatment a proper examination under an anæsthetic should be made, an evacuating tube and aspirator being used if necessary, when, if a stone be discovered, it may be crushed and removed unless for any special reason it be thought desirable to perform lithotomy. In regard to the operations of castration or vasectomy which are now so often done in the hope of reducing enlargement of the prostate, Mr. Buckston Browne speaks with no uncertain voice. He entirely condemns them. It is forgotten, he says, that prostatic patients have their ups and downs, even under the most favourable circumstances, and he points out that a patient after a bad attack of prostatic retention may be entirely dependent on his catheter for twelve months, and yet may gradually recover all his power. In such a case, if subjected to castration, the result would be put down to the operation. He believes that "castration does no real good in genuine cases of prostatic enlargement," and he knows that it is fraught with grave dangers. "Many patients become insane, many become decrepit, and many sink altogether under the operation." What then is to be done? There is only one thing to do for a prostatic patient whose sufferings cannot be cured or mitigated by catheterism and a properly ordered life, and that is to open the bladder suprapubically in order to explore digitally for stone or tumour, and, at any rate, to obtain drainage and rest for that organ. No attempt should be made by the perineum, but the bladder should be opened above the pubes where, however large the prostate may be, the finger can reach every nook and corner of the bladder and deal with whatever may be found. "It is quite curious how often a bad prostatic case will prove to be really a case of calculus, often hidden away in one of the many pouches to which such cases are subject," and when such is the case the removal of the stone may be followed by brilliant results. If a stone be found and the intravesical prostate be not very large the prostate had better be left alone; but if there be much intravesical growth it will become a question for the judgment of the surgeon whether or not it should be removed by prostatectomy. In regard to this operation, which was introduced by the late Mr. McGill, of Leeds, Mr. Buckston Browne holds that it should never be undertaken at the outset of catheter life unless regular auto-catheterism is difficult or well-nigh impossible. Secondly, prostatectomy should never be undertaken as long as the ordinary catheter life is a tolerable one. Thirdly, if catheter life becomes intolerable suprapubic cystotomy should be resorted to, when, if thought desirable, the prostate can be removed. If on the other hand this should seem inadvisable a suprapubic tube may be left in for permanent afterwear with the certainty that the condition of the patient will be considerably improved. Prostatic patients should therefore be recommended to be content with the catheter life as long as it is tolerable, and in the vast majority of cases, with reasonable care, it will remain tolerable into extreme old age—until the end comes probably through other channels.

